# Development of pH-Responsive Hyaluronic Acid-Conjugated Cyclodextrin Nanoparticles for Chemo-/CO-Gas Dual Therapy

**DOI:** 10.3390/pharmaceutics15071818

**Published:** 2023-06-25

**Authors:** Eunsol Lee, Eun Seong Lee

**Affiliations:** 1 Department of Biotechnology, The Catholic University of Korea, 43 Jibong-ro, Bucheon-si 14662, Gyeonggi-do, Republic of Korea; eunsollee13@gmail.com; 2 Department of Biomedical-Chemical Engineering, The Catholic University of Korea, 43 Jibong-ro, Bucheon-si 14662, Gyeonggi-do, Republic of Korea

**Keywords:** pH-responsive γ-cyclodextrin, tumor targeting, paclitaxel, triiron docecacarbonyl, dual therapy

## Abstract

In this study, we fabricated γ-cyclodextrin (γCD)-based nanoparticles (NPs) for dual antitumor therapy. First, γCD (the backbone biopolymer) was chemically conjugated with low-molecular-weight hyaluronic acid (HA; a tumoral CD44 receptor-targeting molecule) and 3-(diethylamino)propylamine (DEAP; a pH-responsive molecule), termed as γCD-(DEAP/HA). The obtained γCD-(DEAP/HA) self-assembled in aqueous solution, producing the γCD-(DEAP/HA) NPs. These NPs efficiently entrapped paclitaxel (PTX; an antitumor drug) and triiron dodecacarbonyl (FeCO; an endogenous cytotoxic gas molecule) via hydrophobic interactions between PTX and FeCO with the unprotonated DEAP molecules in γCD-(DEAP/HA) and a possible host–guest interaction in the γCD rings. The release of PTX and FeCO from the NPs resulted from particle destabilization at endosomal pH, probably owing to the protonation of DEAP in the NPs. In vitro studies using MCF-7 tumor cells demonstrated that these NPs were efficiently internalized by the cells expressing CD44 receptors and enhanced PTX/FeCO-mediated tumor cell apoptosis. Importantly, local light irradiation of FeCO stimulated the generation of cytotoxic CO, resulting in highly improved tumor cell death. We expect that these NPs have potential as dual-modal therapeutic candidates with enhanced antitumor activity in response to acidic pH and local light irradiation.

## 1. Introduction

In the development of stimuli-sensitive drug delivery systems, designing site-specific drug carriers by engineering functional biopolymers has often resulted in increasing the local drug dose or maximizing the drug efficacy at specific locations, thus reducing side effects [1,2,3,4]. In particular, the various functionalities of biopolymers or bioactive materials endow drug carriers with attractive physicochemical reactivity and benefit drug carrier reactions with genetic materials, proteins, and biosignals [2,5,6,7]. Cyclodextrin (CD), a cyclic oligosaccharide, is a known host molecule with a central cavity that allows the inclusion of a guest drug molecule [8,9,10,11]. Remarkably, chemically incorporating functional molecules into CDs is an interesting strategy that strengthens the site-specific bioreaction of CDs, which has often been used to improve drug therapeutic efficacy [10,11,12,13]. Recently, multifunctional CD-based nanoparticles (NPs) have been widely reported for the efficient transportation of antitumor drugs to tumors in response to specific stimuli such as temperature, light, redox, and pH [14,15,16,17,18]. However, such drug carriers have not always shown excellent antitumor effects because they may not overcome the complexities of the cells during the treatment process [16,19,20,21]. Importantly, it has often been reported that when functional drug carriers transport two or more drugs and respond to multiple stimuli, their multifaceted antitumor effect can sometimes enhance therapeutic outcomes more effectively than a single-drug administration system [16,19,20,21,22].

In this study, we report dual-modal therapeutic CD-based NPs that respond to both the slightly acidic pH of endosomes and local light irradiation, resulting in the accelerated delivery of paclitaxel (PTX; an antitumor model drug) and carbon monoxide (CO; a cytotoxic model gas) to the local tumor site. PTX can bind microtubules in tumor cells and effectively inhibit cell mitosis, ultimately inducing cell apoptosis [23,24]. CO gas is a potent chemotherapeutic agent that induces tumor cell death through mitochondrial damage. In addition, the hydrophobic prodrug triiron dodecacarbonyl (FeCO) can be degraded under light irradiation, releasing CO gas [25,26]. Therefore, we entrapped both PTX and FeCO in CD-based NPs. We also hypothesized that the CO gas released from FeCO after light irradiation would provide additional antitumor effects, such as CO gas-mediated mitochondrial damage in tumor cells, which would effectively enhance PTX-mediated cell death. Recent studies have shown that the administration of a combination of multiple drugs with different mechanisms produces a more significant, synergistic antitumor effect and fewer side effects than single-drug therapy [19,20,21,22].

**Figure 1:**
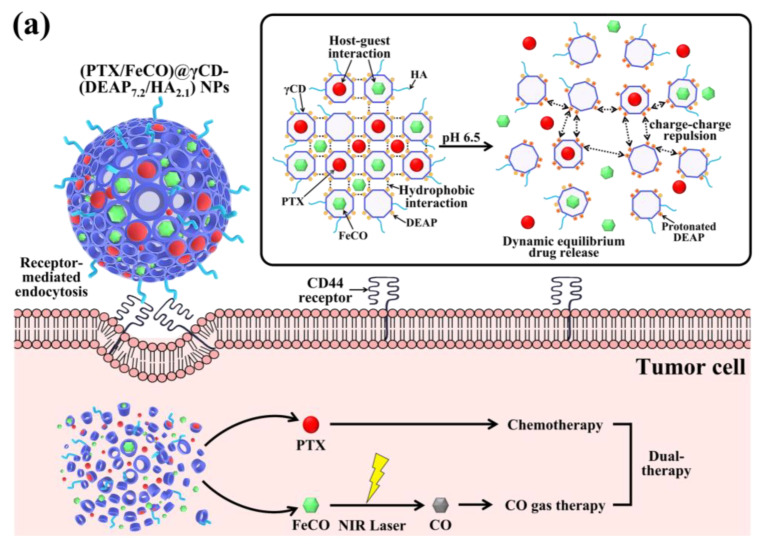

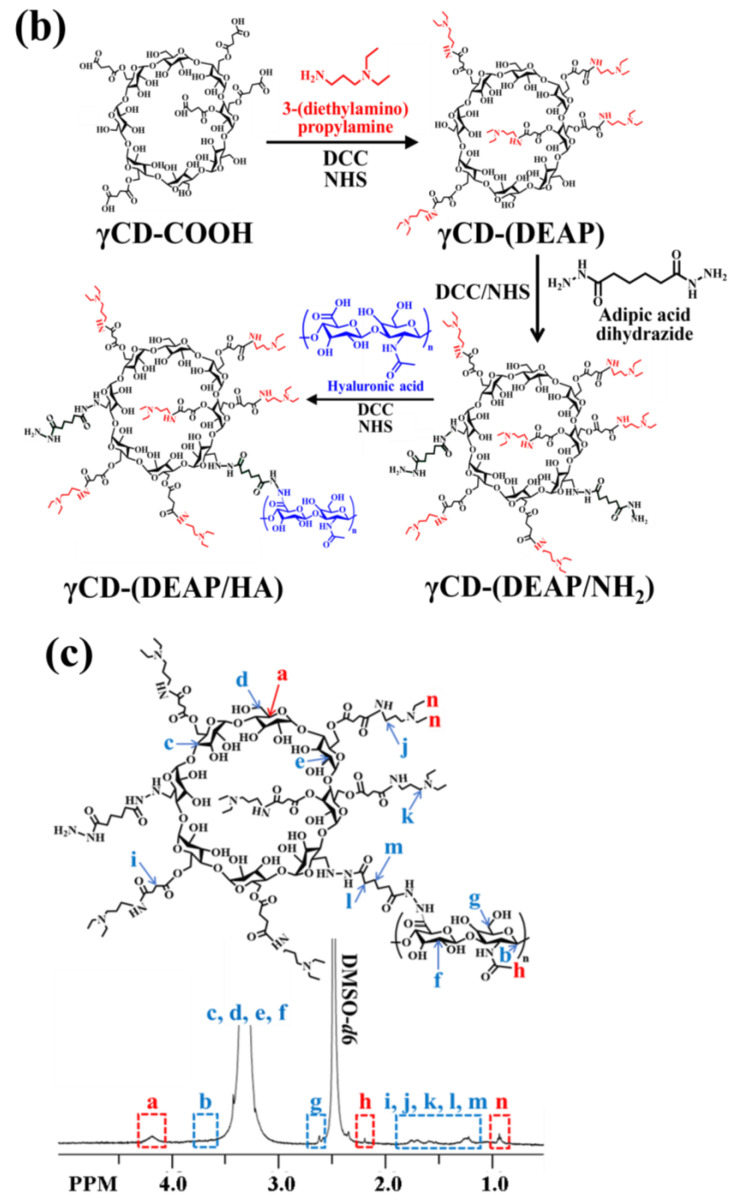
(**a**) Schematic illustration of (PTX/FeCO)@γCD-(DEAP_7.2_/HA_2.1_) NPs. (**b**) Synthesis scheme of γCD-(DEAP/HA). (**c**) ^1^H-NMR spectrum of γCD-(DEAP_7.2_/HA_2.1_). The red font in Figure 1c was used for the comparison of ^1^H-NMR peaks.

To achieve our goal, we fabricated functional CD-based NPs by conjugating γ-cyclodextrin (γCD) with 3-(diethylamino)propylamine (DEAP; a pH-responsive molecule) [17,18,27] and hyaluronic acid (HA; a CD44 receptor-targeting molecule) [28,29,30,31]. The self-assembly of γCD with DEAP and HA resulted in the formation of γCD-(DEAP/HA) NPs, driven by hydrophobic interactions between DEAP moieties and hydrophilic interactions between γCD and HA. These NPs exhibited organized porosity and enabled multiple interactions between the host (γCD) and guest molecules (PTX and FeCO) [9,10,11,32,33], allowing for the encapsulation of these therapeutic agents. Upon selective internalization into CD44 receptor-expressing tumor cells, the protonated DEAP moieties in the NPs, triggered by the acidic endosomal pH, destabilized the NPs and facilitated the rapid release of PTX and CO gas when exposed to light (Figure 1a). Thus, we focused on investigating the physicochemical properties and release profiles of PTX and CO gas, as well as the in vitro antitumor activity of the γCD-(DEAP/HA) NPs to evaluate their pharmaceutical potential.

## 2. Materials and Methods

### 2.1. Materials

γ-Cyclodextrin (γ-CD), succinic anhydride (SA), dimethylsulfoxide (DMSO), N,N’-dicyclohexylcarbodiimide (DCC), 4-dimethylaminopyridine (DMAP), 3-(diethylamino)propylamine (DEAP), hydroxysuccinimide (NHS), triethylamine (TEA), sodium tetraborate, adipic acid dihydrazide (ADH), N-phenylacetic acid (PA), methanol (MeOH), triiron dodecacarbonyl (FeCO), acetonitrile (HPLC grade), deionized water (HPLC grade), sodium azide, tween 80, hemoglobin (Hb), sodium dithionite, 4′,6-diamidino-2-phenylindole dihydrochloride (DAPI), and formaldehyde were purchased from Sigma-Aldrich (St. Louis, MO, USA). Sodium hyaluronate (HA, M_w_ = 4.8 kDa) was purchased from Lifecore Biomedical Inc. (Chaska, MN, USA). Paclitaxel (PTX) was purchased from Samyang Biopharm (Seoul, Republic of Korea). RPMI-1640, fetal bovine serum (FBS), penicillin, streptomycin, trypsin, and ethylene diamine tetra-acetic acid (EDTA) were purchased from Welgene Inc. (Seoul, Republic of Korea). Chlorin e6 (Ce6) was purchased from Frontier Scientific Inc. (Logan, UT, USA). Wheat Germ Agglutinin Alexa Fluor^®^ 488 conjugate (WGA-Alexa Fluor^®^ 488) was purchased from Life Technologies (Carlsbad, CA, USA). Cell Counting Kit-8 (CCK-8) was purchased from Dojindo Molecular Technologies Inc. (Rockville, MD, USA).

### 2.2. Preparation of γCD-(DEAP/HA)

γCD (300 mg) reacted with SA (644 mg) in DMSO (15 mL) containing DCC (530 mg) and DMAP (270 mg) for 3 days, producing carboxylated γCD (γCD-COOH). The resulting solution was dialyzed using a dialysis membrane (Spectra/Por^®^ MWCO 1 kDa) for 2 days in fresh DMSO and 2 days in distilled water to remove the non-reacted chemicals and then lyophilized [17,27]. Next, γCD-COOH (100 mg) reacted with DEAP (200 mg or 150 mg) in DMSO (15 mL) containing DCC (300 mg), NHS (250 mg), and TEA (1 mL) for 3 days, producing DEAP-conjugated γCD [γCD-(DEAP)]. The resulting solution was dialyzed using a dialysis membrane (Spectra/Por^®^ MWCO 1 kDa) for 2 days in fresh DMSO and 2 days in 5 mM sodium tetraborate solution and then lyophilized [17,27]. Then, γCD-(DEAP) (100 mg) reacted with ADH (174 mg) in DMSO (15 mL) containing DCC (770 mg), NHS (434 mg), and TEA (1 mL) for 2 days [17,27]. The resulting solution further reacted with preactivated HA [150 mg, preactivated for 1 day in DMSO (15 mL) containing DCC (770 mg), NHS (434 mg), and TEA (0.5 mL)] for 3 days, producing γCD-(DEAP/HA) (Figure 1b). The resulting solution was dialyzed in fresh DMSO for 2 days and 5 mM sodium tetraborate solution for 2 days using a dialysis membrane (Spectra/Por^®^ MWCO 3.5 kDa) and then lyophilized.

γCD-(PA/HA) were synthesized as a pH-insensitive control group. Here γCD-COOH (300 mg) reacted with pH-insensitive PA (644 mg) in DMSO (15 mL) containing DCC (530 mg) and DMAP (270 mg) for 5 days. The resulting solution was dialyzed to remove non-reacted chemicals using a dialysis membrane (Spectra/Por^®^ MWCO 1 kDa) for 2 days in DMSO and 2 days in distilled water, followed by lyophilization [17,27]. Furthermore, γCD-PA (100 mg) reacted with ADH (174 mg) in DMSO (15 mL) containing DCC (770 mg), NHS (434 mg), and TEA (1 mL) for 2 days. The resulting solution further reacted with preactivated HA [150 mg, preactivated for 1 day in DMSO (15 mL) containing DCC (770 mg), NHS (434 mg), and TEA (0.5 mL)] for 3 days to prepare γCD-(PA/HA). The resulting solution was dialyzed using a dialysis membrane (Spectra/Por^®^ MWCO 3.5 kDa) and then lyophilized.

The chemical structure of each γCD derivative was analyzed using proton nuclear magnetic resonance (^1^H-NMR) analysis (300 MHz NMR Spectrometer, Bruker, Germany) [17,18,27].

### 2.3. Characterization of γCD-Based NPs

γCD-based polymers (100 mg) dissolved in DMSO (10 mL) were dialyzed using a dialysis membrane (Spectra/Por^®^ MWCO 2 kDa), producing γCD-based NPs [17,18]. The morphologies of γCD-based NPs with and without laser irradiation at pH 7.4 and 6.5 were monitored using field emission scanning electron microscopy (FE-SEM, Hitachi S-4800, Nagano, Japan) [17,18,29,30]. Prior to measurement, the NPs (0.1 mg) were stabilized in 150 mM PBS (pH 7.4 or 6.5, 1 mL) for 4 h and irradiated with a laser at an intensity of 0 W/cm^2^ or 1 W/cm^2^ for 10 min. In addition, the average particle size and zeta potentials of NPs (0.1 mg) in 150 mM PBS (pH 7.4 or 6.5, 1 mL) were measured using a Zetasizer 3000 instrument (Malvern Instruments, Malvern, UK) [17,18,29,30]. Prior to each measurement, the NPs (0.1 mg/mL) were stabilized for 4 h and irradiated with a laser at an intensity of 0 W/cm^2^ or 1 W/cm^2^ for 10 min.

### 2.4. PTX and FeCO Loading

γCD-based polymers (100 mg) dissolved in a DMSO (10 mL)/MeOH (10 mL) co-solvent containing PTX (100 mg) and FeCO (80 mg) were stirred for 1 day under dark conditions [17,34]. The resulting solution was dialyzed against DMSO, ultracentrifuged at 20,000 rpm for 30 min, and then lyophilized. Unencapsulated PTX and FeCO were removed by filtration through a 0.2 μm pore size filter [17,18]. The amount of encapsulated PTX in the NPs was confirmed using high-performance liquid chromatography (HPLC, Waters, MA, USA) analysis. For HPLC analysis, the solution was transferred to the mobile phase (acetonitrile/deionized water, 60/40, vol.%), separated using a CAPCELL PAK C_18_ column (250 × 4.6 mm, 5 μm, Shiseido Co. Ltd., Tokyo, Japan) at 25 °C, and detected at 227 nm [27]. The PTX or FeCO loading efficiency (%) was calculated as a weight percentage of the PTX or FeCO dose loaded into the NPs to the initial PTX or FeCO fed dose. The PTX or FeCO loading content (%) was calculated as the weight percentage of the encapsulated PTX or FeCO dose to the total amount in the harvested NPs [27,34,35].

### 2.5. In Vitro PTX and CO Release

NPs (with an equivalent PTX concentration of 1 mg/mL or an equivalent FeCO concentration of 100 μg/mL) were dispersed in PBS (150 mM, pH 7.4 or 6.5, 1 mL) and irradiated with a laser at 0 W/cm^2^ or 1 W/cm^2^ for 10 min. The resulting NPs were added to a dialysis membrane (Spectra/Por^®^ MWCO 20 kDa) and immersed in PBS (150 mM, pH 7.4 or 6.5, 15 mL) containing 0.01 wt.% sodium azide and 3% Tween 80 [27]. The membranes were incubated in a mechanical shaking bath (100 rpm) at 37 °C for 48 h. A sample of the solution outside of the dialysis bag was removed at specified times and replaced with fresh PBS. The amount of PTX released from the NPs was analyzed using an HPLC instrument [27]. Next, to confirm the CO release profiles from the NPs at pH 7.4~6.5 with and without laser irradiation, the carboxyhemoglobin (HbCO) method was used. Briefly, sodium-dithionite-treated Hb was mixed with light-irradiated NPs in PBS (150 mM, pH 7.4 or 6.5) at 25 °C for 30 min [34,35]. The ultraviolet/visible (UV/Vis) absorption spectra (350–600 nm) of the solutions were monitored at each incubation time using a Cary 1E UV/visible spectrophotometer (Varian Inc., Palo Alto, CA, USA), and the conversion of Hb to HbCO was quantified using Beer–Lambert’s law to calculate the CO release from the NPs [34,35].

### 2.6. Cell Culture

Human breast carcinoma MCF-7 (CD44 receptor-positive) or BT-474 (CD44 receptor-negative) cells were obtained from the Korean Cell Line Bank (Seoul, Republic of Korea) and cultured in RPMI-1640 medium containing 10% FBS and 1% penicillin–streptomycin at 5% CO_2_ and 37 °C. Prior to in vitro cell tests, the cells (1 × 10^6^ cells/mL) in a monolayer state were harvested with 0.25% (*w*/*v*) trypsin and 0.03% (*w*/*v*) EDTA solution, seeded into a 96-well culture plate, and cultured in an RPMI-1640 medium for 24 h [17,18,27,29,30].

### 2.7. In Vitro Cellular Uptake

To evaluate the cellular uptake of NPs, NPs were labeled with the fluorescent dye Ce6. Briefly, γCD-based polymers (300 mg) reacted with Ce6 [200 mg, preactivated with ADH (50 mg) for 8 h in DMSO (10 mL) containing DCC (200 mg), NHS (100 mg), and TEA (0.5 mL)] in DMSO (10 mL) containing DCC (100 mg), NHS (50 mg), and TEA (1 mL) for 5 days. To remove unreacted chemicals, the resulting solution was dialyzed using a dialysis membrane (Spectra/Por^®^ MWCO 1 kDa) for 2 days in DMSO and 2 days in 5 mM sodium tetraborate solution, followed by lyophilization [18]. Here Ce6 conjugation to γCD-based polymers was confirmed by ^1^H-NMR analysis [17,18]. We fabricated NPs using fluorescent Ce6 dye-labeled γCD, according to the method mentioned above. The resulting NPs (at an equivalent Ce6 concentration of 10 μg/mL) or free Ce6 (10 μg/mL) were incubated with tumor cells at 37 °C for 4 h. The treated cells were washed with fresh PBS (pH 7.4) three times and analyzed using a FACSCalibur^TM^ flow cytometer (FACSCanto II, Becton Dickinson, Franklin Lakes, NJ, USA). In addition, the cellular distribution of the NPs was visualized in cells stained with DAPI and WGA-Alexa Fluor^®^488 using a confocal laser scanning microscope (LSM710, Carl Zeiss, Oberkochen, Germany) and a hyperspectral camera (CytoViva, Auburn, AL, USA) [17,18].

### 2.8. In Vitro Cytotoxicity

The NPs (at an equivalent PTX concentration of 10 μg/mL or an equivalent FeCO concentration of 6.75 μg/mL) or free PTX (10 μg/mL) were incubated with MCF-7 or BT-474 cells at 37 °C for 24 h. Since free FeCO (as a control) was highly insoluble in the cell medium, we added FeCO (10 mg) into γCD solution (10 wt.%), preparing FeCO-inclused γCD (FeCO@γCD, equivalent to FeCO 6.75 μg/mL), and tested its cytotoxicity for MCF-7 or BT-474 cells at 37 °C for 24 h. The treated cells were washed three times and irradiated with an NIR laser at 0 W/cm^2^ or 0.5 W/cm^2^ intensity for 10 min [17,18,34,35]. The laser-irradiated cells were washed three times and further incubated at 37 °C for 12 h in fresh RPMI-1640. The cell viability was measured using a CCK-8 assay [17,18,34,35]. In addition, the tumor cells were incubated with the NPs (1–100 μg/mL) without PTX and FeCO in an RPMI-1640 medium (pH 7.4) at 37 °C for 24 h to evaluate the original toxicity of the NPs [17,18,34,35].

### 2.9. Statistical Evaluation

All experiment results were analyzed using Student’s *t*-test or ANOVA test with *p* < 0.01 (**) as a significance level [29,30].

## 3. Results and Discussion

### 3.1. Preparation of γCD-(DEAP/HA) NPs

First, we synthesized a polymer in which DEAP and HA were conjugated to γCD to control the release of PTX and FeCO (Figure 1a). Briefly, the DEAP moiety (a pH-responsive agent) [17,27] was conjugated to carboxylated γCD (γCD-COOH) using DCC and NHS in DMSO and then coupled with HA (a CD44-targeting hydrophilic biopolymer) [28,29,30], producing γCD-(DEAP/HA) (Figure 1b). The resulting polymer [γCD-(DEAP/HA)] was characterized using ^1^H-NMR and it was found that the molar conjugation ratios of DEAP and HA to γCD were 7.2 and 2.1, respectively, hereafter denoted γCD-(DEAP_7.2_/HA_2.1_), which were estimated from the analysis of the integration ratios of the peaks at δ 0.90 ppm (-C**H**_3_ from DEAP), δ 2.43 ppm (-COC**H**_3_ from HA), and δ 4.20 ppm (-C**H**- from γCD) (Figure 1c). We also synthesized γCD-(DEAP/HA) with different DEAP conjugation ratios. γCD-(DEAP_3.4_/HA_2.1_) (Figure S1) was synthesized to evaluate the effect of DEAP according to the conjugation ratio of DEAP to γCD. Furthermore, γCD with HA and pH-nonresponsive PA [γCD-(PA/HA)] was synthesized for use as a control. The ^1^H-NMR analysis showed that the molar conjugation ratios of PA and HA to γCD were 4.2 and 2.0, respectively, thereafter denoted γCD-(PA_4.2_/HA_2.0_), which was estimated from the analysis of the integration ratios of the peaks at δ 7.20 ppm (-C**H**- from PA), δ 2.43 ppm (-COC**H**_3_ from HA), and δ 4.20 ppm (-C**H**- from γCD) (Figure S2). As an additional control group, γCD with pH-nonresponsive PA (γCD-PA) was also synthesized. The ^1^H-NMR analysis showed that the molar conjugation ratio of PA to γCD was 4.2, thereafter denoted γCD-(PA_4.2_) (Figure S3).

Next, we encapsulated PTX and FeCO (as antitumor model drugs) into γCD-(DEAP/HA) NPs via a dialysis method [17,18]. Here γCD with unprotonated DEAP (pK_b_ ~ 6.8) self-assembled at pH 7.4 to form a porous γCD core, probably owing to hydrophobic interactions between unprotonated DEAP moieties, while HA segments self-assembled into a hydrophilic shell. Importantly, PTX and FeCO were embedded in γCD-(DEAP/HA) NPs, probably owing to hydrophobic interactions between PTX and FeCO with unprotonated DEAP moieties and possible host–guest interactions in the γCD rings (Figure 1a). As a result, the loading efficiencies of PTX (%) in γCD-(DEAP_7.2_/HA_2.1_) NPs, γCD-(DEAP_3.4_/HA_2.1_) NPs, γCD-(PA_4.2_/HA_2.0_) NPs, and γCD-(PA_4.2_) NPs were found to be 51.5 ± 2.3, 49.5 ± 2.7, 50.5 ± 3.2, and 52.0 ± 1.4%, respectively. The loading contents of PTX in γCD-(DEAP_7.2_/HA_2.1_) NPs, γCD-(DEAP_3.4_/HA_2.1_) NPs, γCD-(PA_4.2_/HA_2.0_) NPs, and γCD-(PA_4.2_) NPs were 27.6 ± 2.4, 26.8 ± 1.6, 27.5 ± 3.1, and 27.7 ± 2.9%, respectively. Furthermore, the loading efficiencies of FeCO (%) in γCD-(DEAP_7.2_/HA_2.1_) NPs, γCD-(DEAP_3.4_/HA_2.1_) NPs, γCD-(PA_4.2_/HA_2.0_) NPs, and γCD-(PA_4.2_) NPs were 43.1 ± 3.7, 42.5 ± 2.5, 40.0 ± 3.3, and 43.1 ± 2.7%, respectively. Similarly, the loading contents of FeCO in γCD-(DEAP_7.2_/HA_2.1_) NPs, γCD-(DEAP_3.4_/HA_2.1_) NPs, γCD-(PA_4.2_/HA_2.0_) NPs, and γCD-(PA_4.2_) NPs were 18.5 ± 2.6, 18.4 ± 2.0, 17.4 ± 1.9, and 18.4 ± 2.2%, respectively. However, when the pH of the environment becomes slightly acidic (i.e., endosomal pH), DEAP protonation destabilizes the NPs, weakening the host–guest equilibrium in γCD and thereby accelerating the release of PTX and FeCO [9,10,27,32,33]. In addition, under NIR irradiation, FeCO can be converted into CO gas that attacks the mitochondria of tumor cells (Figure 1a). Based on this hypothesis, we focused on identifying the physicochemical properties of γCD-based NPs and their in vitro antitumor efficacy.

### 3.2. Characterization of γCD-(DEAP/HA) NPs

Figure S4a shows the particle size and particle morphology of γCD-based NPs at pH 7.4 (normal pH) and pH 6.5 (endosomal pH). At pH 7.4, the NPs had an almost spherical shape, but at pH 6.5, the NPs with pH-responsive DEAP moieties [γCD-(DEAP_7.2_/HA_2.1_) and γCD-(DEAP_3.4_/HA_2.1_)] became unstable. In addition, under laser irradiation at an intensity of 1 W/cm^2^ for 10 min, the morphological changes in the NPs were not significant as shown in Figure S4b. These results indicated that the CO gas generated from FeCO under light irradiation could be easily released from the γCD rings without significantly affecting the structure of the NPs.

**Figure 2:**
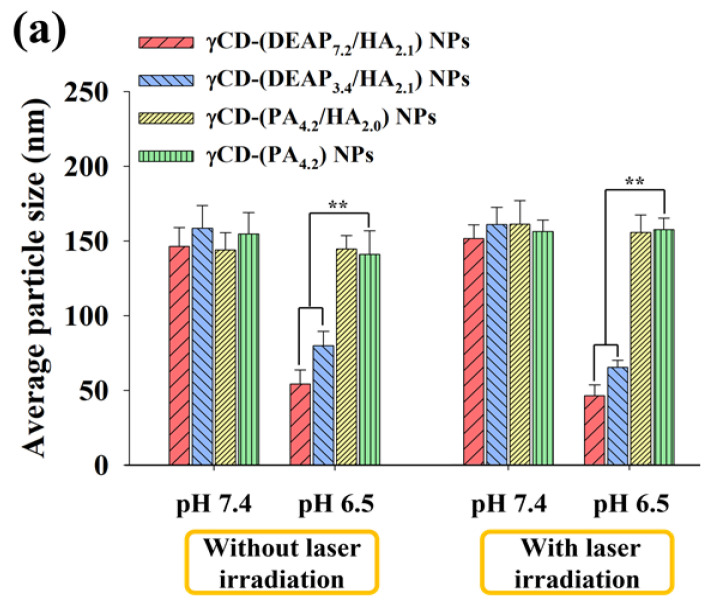

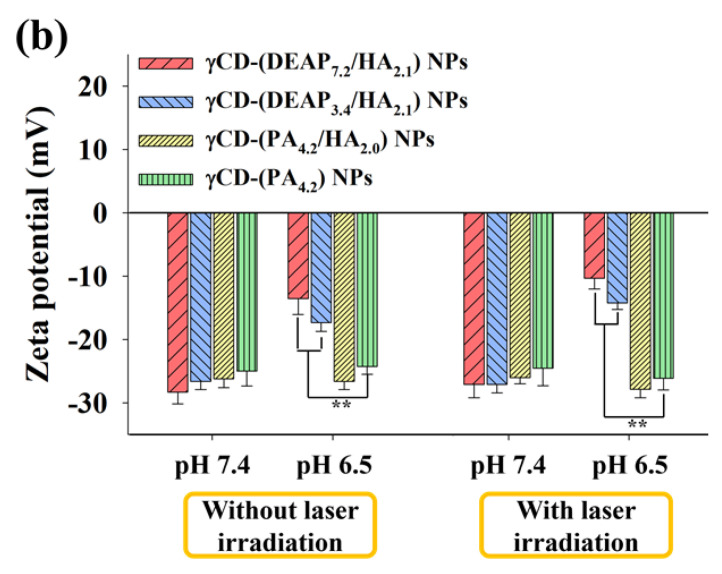
(**a**) Particle size distribution and (**b**) zeta potential values of γCD-(DEAP_7.2_/HA_2.1_) NPs, γCD-(DEAP_3.4_/HA_2.1_) NPs, γCD-(PA_4.2_/HA_2.0_) NPs, and γCD-(PA_4.2_) NPs at pH 7.4 and 6.5 with or without NIR laser irradiation (808 nm, 1 W/cm^2^, 10 min) [n = 3, as multiple experiments, ** *p* < 0.01 compared with γCD-(PA_4.2_) NPs at pH 7.4 or 6.5].

Figure 2a shows that the particle size of γCD-(DEAP_7.2_/HA_2.1_) NPs decreased from 146 nm at pH 7.4 to 54 nm at pH 6.5, probably owing to DEAP protonation at pH 6.5 [17,27,30,31]. In γCD-(DEAP_7.2_/HA_2.1_) NPs, the particle size reduction at pH 6.5 was much greater than that of γCD-(DEAP_3.4_/HA_2.1_) NPs, revealing the effect of a high DEAP conjugation ratio. In addition, under 1 W/cm^2^ laser irradiation for 10 min, the changes in the particle size of all the NPs were not significant.

Figure 2b shows that the zeta potential of γCD-(DEAP_7.2_/HA_2.1_) NPs increased from -28 mV at pH 7.4 to −13 mV at pH 6.5, probably owing to DEAP protonation at pH 6.5 [17,27,30,31]. In addition, the zeta potential changes of γCD-(PA_4.2_/HA_2.0_) NPs and γCD-(PA_4.2_) NPs as control groups were not significant, which is similar to their particle size results with no significant difference between pH 6.5 and 7.4 (Figure 2a and Figure S4).

### 3.3. In Vitro Release Profiles of γCD-(DEAP/HA) NPs

**Figure 3:**
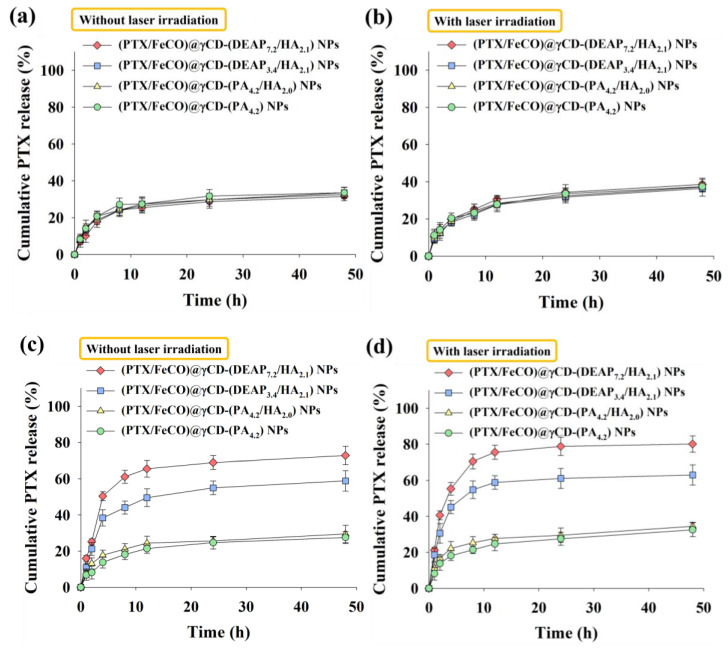
Cumulative PTX release profiles from each type of γCD NP (**a**) without or (**b**) with NIR laser irradiation at pH 7.4. Cumulative PTX release profiles from each type of γCD NP (**c**) without or (**d**) with NIR laser irradiation at pH 6.5 (808 nm, 1 W/cm^2^, 10 min, n = 3, as multiple experiments).

**Figure 4:**
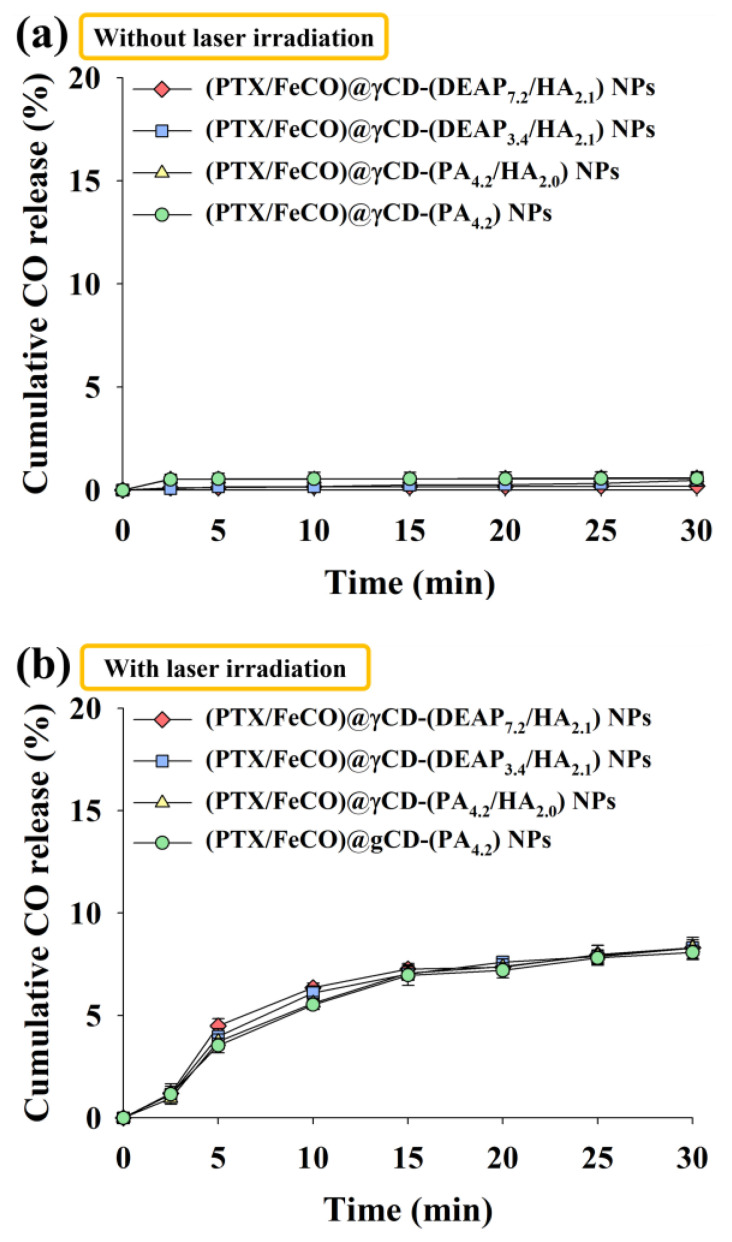
Cumulative CO release profiles from each type of γCD NP for 30 min at light intensities of (**a**) 0 W/cm^2^ and (**b**) 1 W/cm^2^ using an NIR laser (n = 3, as multiple experiments). The cumulative release of CO (%) was calculated by determining the ratio between the CO values released from NPs and the CO values that would theoretically be released from FeCO.

We investigated the drug release behaviors from the γCD-based NPs at pH 7.4 and 6.5 (Figure 3 and Figure 4) [17,18,27,30,31]. First, the cumulative PTX release from γCD-based NPs at pH 7.4 was approximately 30 wt.% in 24 h, revealing a conventional passive drug release pattern (Figure 3a) [27,30,31]. Under light irradiation (Figure 3b), the γCD-based NPs at pH 7.4 showed similar PTX release as in the case without light irradiation. However, the cumulative PTX release from the γCD-(DEAP_7.2_/HA_2.1_) NPs and γCD-(DEAP_3.4_/HA_2.1_) NPs at pH 6.5 averaged 69 and 55 wt.% over 24 h, respectively, probably owing to the DEAP protonation-mediated destabilization of the NPs [17,27,30,31]. In addition, under light irradiation (Figure 3d), the γCD-(DEAP_7.2_/HA_2.1_) NPs and γCD-(DEAP_3.4_/HA_2.1_) NPs at pH 7.4 showed similar PTX release as that in the case without light irradiation (Figure 3c).

We also evaluated the amount of CO gas generated from FeCO under light irradiation (Figure 4). Here the amount of CO gas was calculated using the HbCO method [34,35]. First, the amount of CO gas released from the nonirradiated γCD-based NPs was not significant (Figure 4a). However, the amount of CO gas released from NPs under light irradiation significantly increased regardless of the sample type, suggesting that the generated CO molecules could sufficiently pass through the γCD rings. As a result, our system also signified that NIR could serve as a key stimulus for specific substance release, as demonstrated in a recently published NIR-triggered DOX releasing nanocluster system [36].

### 3.4. In Vitro Cellular Internalization

**Figure 5:**
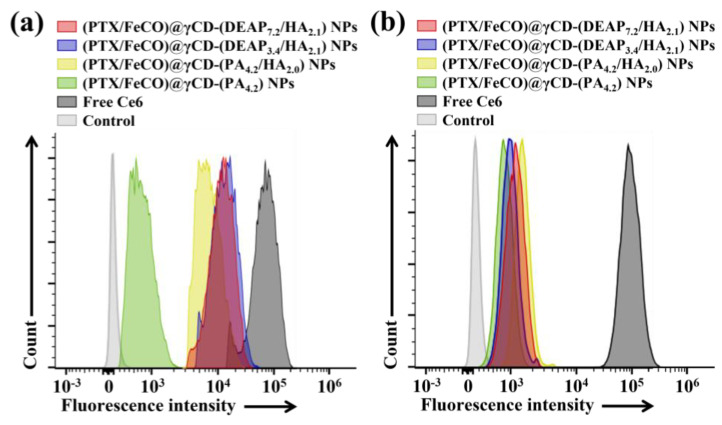
Flow cytometry analysis of (**a**) MCF-7 cells and (**b**) BT-474 cells treated with each type of NP (equivalent to 10 μg/mL Ce6) or free Ce6 (10 μg/mL) for 4 h at 37 °C.

**Figure 6:**
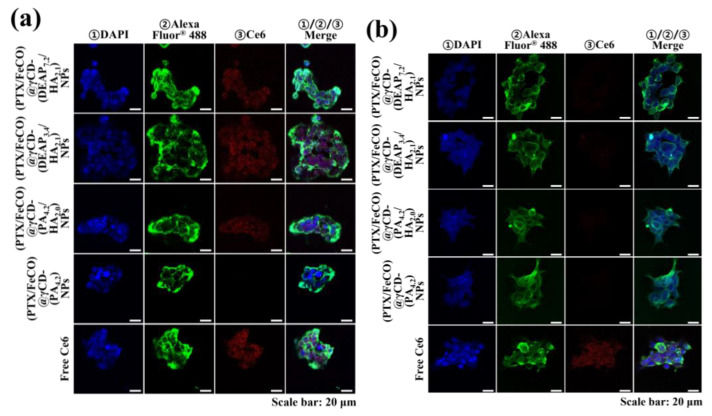
Confocal images of (**a**) MCF-7 cells and (**b**) BT-474 cells treated with each type of NP (equivalent to 10 μg/mL Ce6) or free Ce6 (10 μg/mL) for 4 h at 37 °C. The treated cells were stained with ① DAPI and ② WGA-Alexa Fluor^®^ 488. In addition, ③ florescent Ce6 was located in NPs.

**Figure 7:**
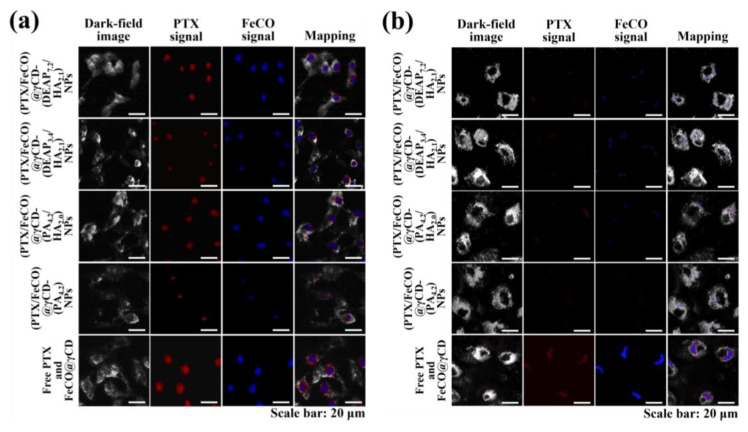
Hyperspectral images of (**a**) MCF-7 cells and (**b**) BT-474 cells treated with each type of NP (equivalent to 10 μg/mL PTX and 6.75 μg/mL FeCO), FeCO@ γCD (equivalent to 6.75 μg/mL PTX), or free PTX (10 μg/mL) for 4 h at 37 °C.

The cellular uptake of γCD-based NPs by MCF-7 (CD44 receptor-positive) [37,38] and BT-474 (CD44 receptor-negative) [29] tumor cells was evaluated using flow cytometry and confocal laser scanning microscopy. Before testing, each γCD-based NP was labeled with the fluorescent dye Ce6; Ce6 was bound at an average molar ratio of 1.1 molecules per γCD unit [17,18]. Figure 5a shows that the γCD-based NPs with HA exhibited higher fluorescence intensity in MCF-7 cells than the γCD-based NPs without HA, revealing the HA ligand in the γCD-based NPs mediated extensive CD44 receptor interactions. However, all NPs showed relatively decreased cellular uptake by BT-474 cells that did not express the CD44 receptor (Figure 5b). In addition, free Ce6, which had no cell-specific interaction abilities, interacted well with both MCF-7 and BT-474 cells. It is known that free Ce6 is well absorbed even by normal cells. Moreover, the confocal microscopy images supported that the γCD-based NPs with HA had higher cellular uptake (i.e., a higher Ce6 signal) in MCF-7 cells than in BT-474 cells, suggesting that γCD-based NPs with HA could effectively target tumor cells with specific CD44 receptors (Figure 6) [29,37,38]. We also evaluated the cellular uptake of PTX and FeCO delivered by γCD-based NPs using a hyperspectral camera [17,18]. The resulting images demonstrated that the γCD-based NPs with HA enabled higher cellular uptake of PTX and FeCO in MCF-7 cells than in BT-474 cells (Figure 7a,b), indicating that the NPs successfully delivered PTX and FeCO into MCF-7 cells.

### 3.5. In Vitro Cell Cytotoxicity

**Figure 8:**
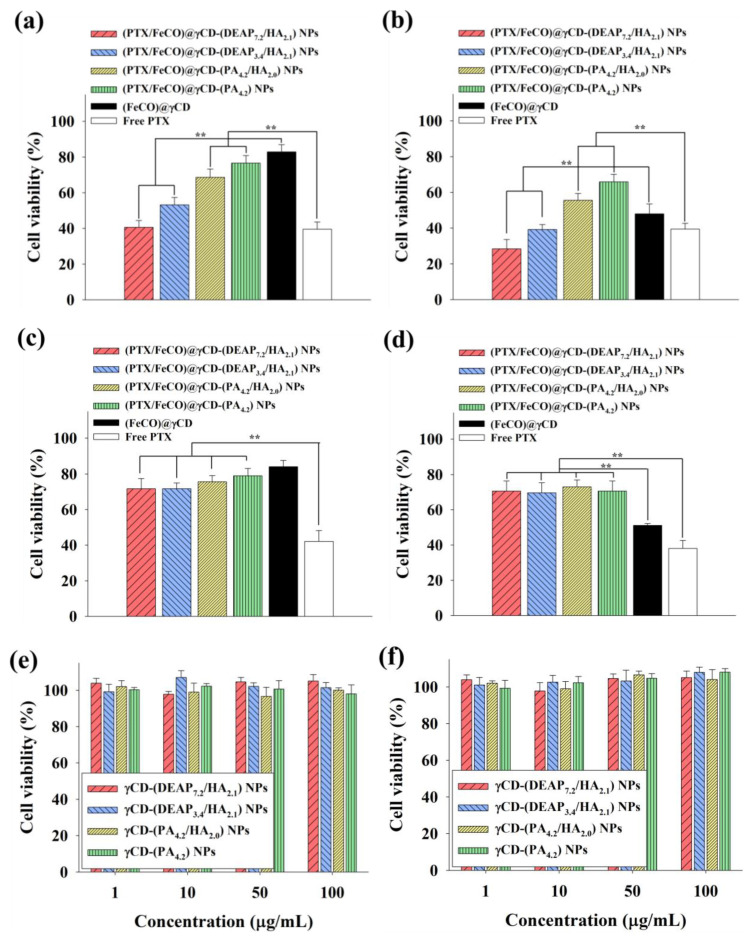
Cell viabilities of MCF-7 cells treated with each type of NP (equivalent to 10 μg/mL PTX and 6.75 μg/mL FeCO), (FeCO)@γCD (equivalent to 6.75 μg/mL FeCO), or free PTX (10 μg/mL) for 24 h at 37 °C (**a**) without or (**b**) with NIR laser irradiation (808 nm, 0.5 W/cm^2^, 10 min). Cell viabilities of BT-474 cells treated with each type of NP (equivalent to 10 μg/mL PTX and 6.75 μg/mL FeCO), (FeCO)@γCD (equivalent to 6.75 μg/mL FeCO), or free PTX (10 μg/mL) for 24 h at 37 °C (**c**) without or (**d**) with NIR laser irradiation (808 nm, 0.5 W/cm^2^, 10 min) [n = 7, as multiple experiments, ** *p* < 0.01 compared with the (FeCO)@γCD or ** *p* < 0.01 compared with the free PTX]. Cell viabilities of (**e**) MCF-7 cells and (**f**) BT-474 cells treated with each type of NP (1–100 μg/mL, without PTX and FeCO) at pH 7.4 for 24 h (n = 7, as multiple experiments).

Figure 8a shows that the γCD-based NPs with HA and pH-responsive DEAP moieties [(PTX/FeCO)@γCD-(DEAP_7.2_/HA_2.1_) and (PTX/FeCO)@γCD-(DEAP_3.4_/HA_2.1_)] exhibited a relatively increased MCF-7 tumor cell killing effect compared with the control groups, probably owing to their CD44 receptor-targeting ability (Figure 5, Figure 6 and Figure 7) and endosomal pH-responsive drug release (Figure 3) [17,27,30,31]. Under light irradiation, γCD-based NPs with HA and pH-responsive DEAP moieties produced excellent MCF-7 cell death, probably owing to the production of mitochondrial-toxic CO (Figure 8b) [25,26,34,35]. Figure S5 demonstrates that CO exerted its cytotoxicity by affecting mitochondrial function and promoting cell death [25,26,34,35]. Notably, the combination of PTX and FeCO for tumor treatment seemed to improve the therapeutic effect on tumor cell death due to their different mechanisms. These aggressive antitumor mechanisms of PTX and CO might be applicable to improve preclinical/clinical antitumor efficacy [16,19,20,21,22]. Figure 8c,d show that the γCD-based NPs with HA relatively less effectively inhibited BT-474 cell proliferation, suggesting that (PTX/FeCO)@γCD-(DEAP_7.2_/HA_2.1_) NPs and (PTX/FeCO)@γCD-(DEAP_3.4_/HA_2.1_) NPs only effectively suppressed MCF-7 cells (CD44 receptor-positive). In addition, the γCD-based NPs with DEAP_7.2_ showed relatively higher antitumor activity than those with DEAP_3.4_, revealing that the γCD-based NPs with a relatively high DEAP conjugation ratio improved the drug release rate (Figure 3) and resulted in an increased antitumor effect. Furthermore, we evaluated the toxicity of the γCD-based NPs without PTX and FeCO and found that they were not significantly toxic to MCF-7 and BT-474 cells (Figure 8e,f) [17,18,29,30].

## 4. Conclusions

In this study, γCD with a pH-responsive DEAP molecule and tumor CD44 receptor-targeting HA was chemically synthesized, and these functional polymers were used to fabricate NPs that effectively delivered PTX and CO gas to tumor sites. These NPs reacted sensitively to endosomal pH and rapidly released PTX and CO gas through the γCD pores or dissociated γCD molecules. This dual-modal therapy exhibited improved antitumor activity compared with the conventional single-drug formulation. This approach based on a functional polysaccharide conjugate is expected to be a novel antitumor drug delivery candidate.

## Data Availability

Not applicable.

## Supplementary Materials

The following supporting information can be downloaded at https://www.mdpi.com/article/10.3390/pharmaceutics15071818/s1: Figure S1: ^1^H-NMR peaks of γCD-(DEAP_3.4_/HA_2.1_); Figure S2: ^1^H-NMR peaks of γCD-(PA_4.2_/HA_2.0_); Figure S3: ^1^H-NMR peaks of γCD-(PA_4.2_); Figure S4: FE-SEM images of NPs at pH 7.4 and 6.5 without laser irradiation and under laser irradiation; Figure S5: Mitochondrial membrane hyperpolarization of MCF-7 or BT-474 cells treated with (PTX/FeCO)@γCD-(DEAP_7.2_/HA_2.1_) NPs. In brief, the MCF-7 or BT-474 tumor cells were incubated with (PTX/FeCO)@γCD-(DEAP_7.2_/HA_2.1_) NPs, with an equivalent FeCO concentration of 6.75 μg/mL, at 37 °C for 4 h. Subsequently, the cells were washed with fresh PBS (pH 7.4) and exposed to 808 nm irradiation at a power density of 0.5 W/cm^2^ for 10 min. Afterward, the resulting cells (1 × 10^6^ cells) were treated with 1,1′,3,3′-tetraethyl-5,5′,6,6′-tetrachloroimidacarbocyanine iodide (JC-1) at a concentration of 0.25 μg/mL for 20 min. The cells were then examined using a fluorescence microscope to analyze the mitochondrial membrane hyperpolarization. The JC-1 dye used in this experiment was purchased from Sigma-Aldrich (St. Louis, MO, USA).
