# Supplementary material for: Development of pH-Responsive Hyaluronic Acid-Conjugated Cyclodextrin Nanoparticles for Chemo-/CO-Gas Dual Therapy
Source: Pharmaceutics. 2023 Jun 25;15(7):1818. doi: 10.3390/pharmaceutics15071818 (PMC10384822; doi:10.3390/pharmaceutics15071818)
Supplement: Supplementary file 1 [file pharmaceutics-15-01818-s001.zip › pharmaceutics-2450470-supplementary.pdf]

Supplementary Data

Development of pH-responsive hyaluronic acid-conjugated cyclodextrin nanoparticles for chemo-/CO-gas dual therapy

Eunsol Lee [1], Eun Seong Lee [1,2,*]

[1]Department of Biotechnology, The Catholic University of Korea, 43 Jibong-ro, Bucheon-si 1462, Gyeonggi-do, Korea; eunsollee13@gmail.com (E.L.); eslee@catholic.ac.kr (E.S.L.)

[2]Department of Biomedical-Chemical Engineering, The Catholic University of Korea, 43 Jibong-ro, Bucheon-si 1462, Gyeonggi-do, Korea; eslee@catholic.ac.kr (E.S.L.)

[*]To whom correspondence should be addressed.

Tel: +82-2-2164-4921, Fax: +82-2-2164-4865

E-mail: eslee@catholic.ac.kr

Supplementary Information

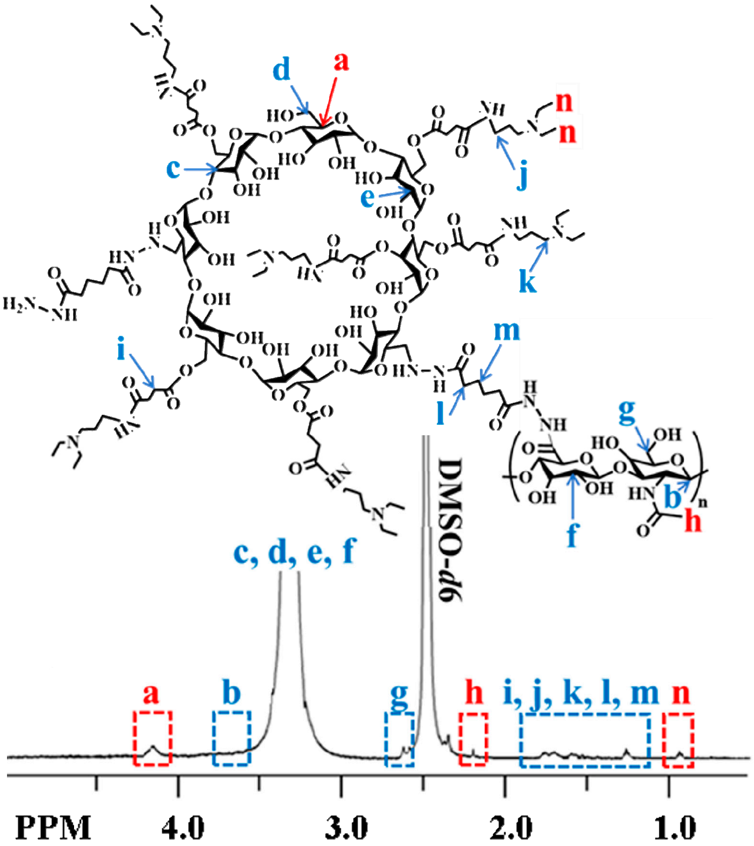

Figure. S1. ^{1}H-NMR peaks of γCD-(DEAP$_{3.4}$/HA$_{2.1}$).

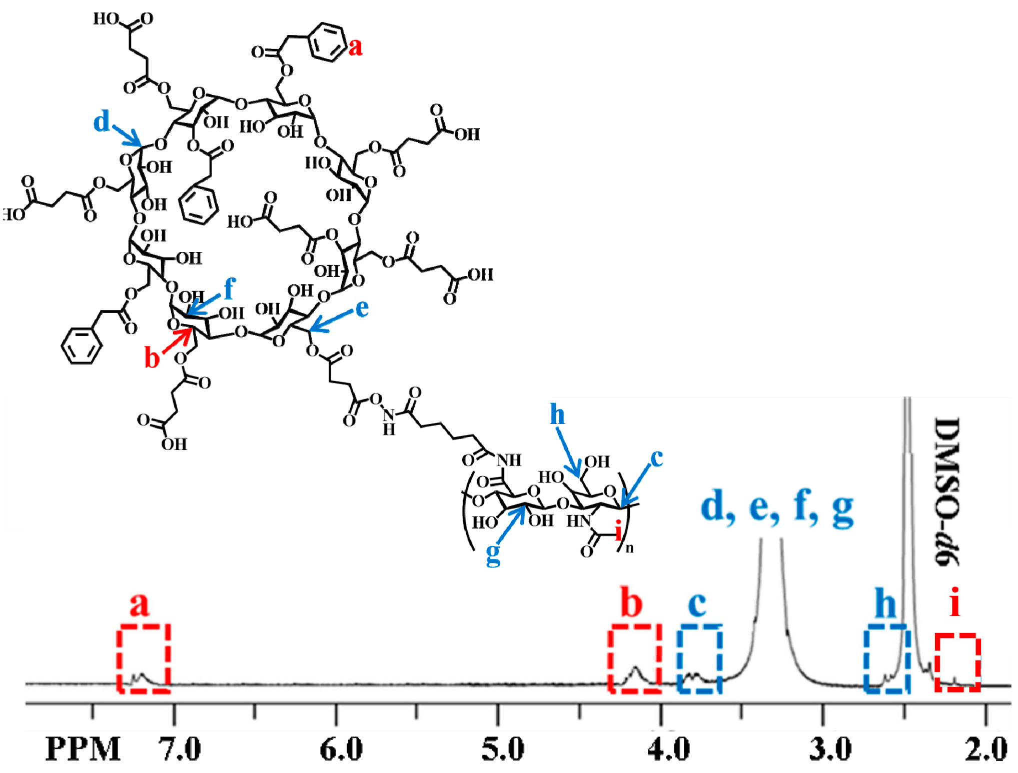

Figure. S2. ^{1}H-NMR peaks of γCD-($PA_{4.2}$/$HA_{2.0}$).

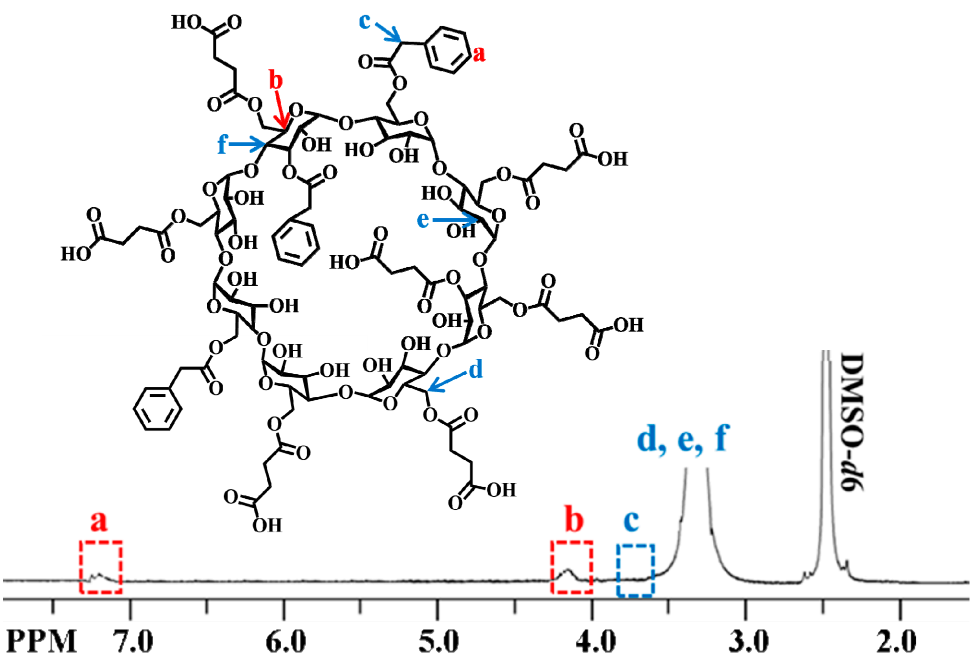

Figure. S3. ^{1}H-NMR peaks of γCD-(PA$_{4.2}$).

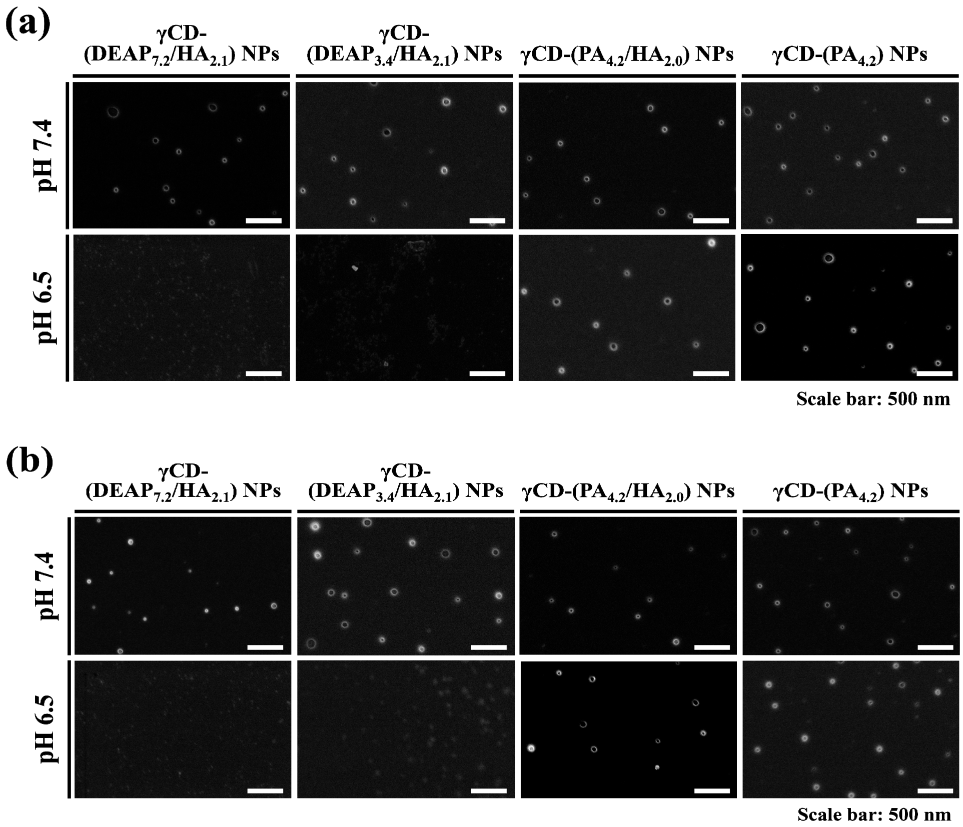

Figure. S4. FE-SEM images of γCD-(DEAP$_{7.2}$/HA$_{2.1}$) NPs, γCD-(DEAP$_{3.4}$/HA$_{2.1}$) NPs, γCD-(PA$_{4.2}$/HA$_{2.0}$) NPs, and γCD-(PA$_{4.2}$) NPs at pH 7.4 and 6.5 (**a**) without laser irradiation and (**b**) under laser irradiation at a light intensity of 1 W/cm^2 for 10 min.

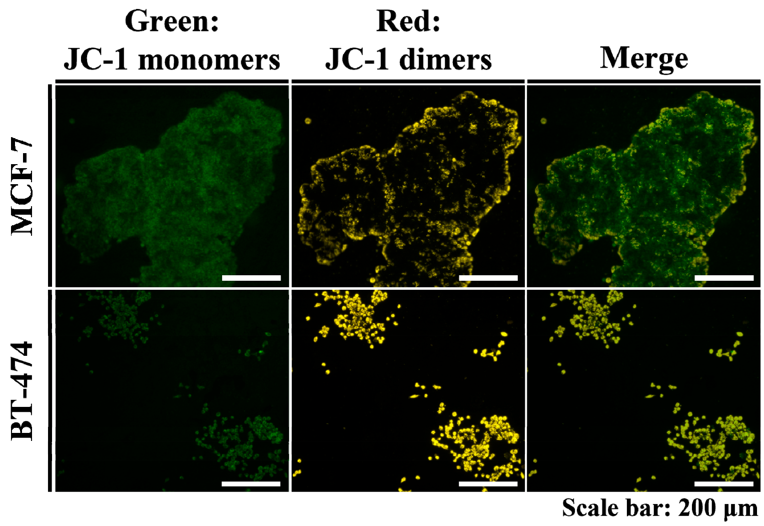

Figure. S5. Mitochondrial membrane hyperpolarization of MCF-7 or BT-474 cells treated with (PTX/FeCO)@γCD-(DEAP$_{7.2}$/HA$_{2.1}$) NPs. In brief, the MCF-7 or BT-474 tumor cells were incubated with (PTX/FeCO)@γCD-(DEAP$_{7.2}$/HA$_{2.1}$) NPs, with an equivalent FeCO concentration of 6.75 µg/mL, at 37 °C for 4 h. Subsequently, the cells were washed with fresh PBS (pH 7.4) and exposed to 808 nm irradiation at a power density of 0.5 W/cm^2 for 10 min. Afterward, the resulting cells (1×10^6 cells) were treated with 1,1',3,3'-tetraethyl-5,5',6,6'-tetrachloroimidacarbocyanine iodide (JC-1) at a concentration of 0.25 µg/mL for 20 min. The cells were then examined using a fluorescence microscope to analyze the mitochondrial membrane hyperpolarization. The JC-1 dye used in this experiment was purchased from Sigma-Aldrich (St. Louis, MO, USA).
